# Where We Stand: Lung Organotypic Living Systems That Emulate Human-Relevant Host–Environment/Pathogen Interactions

**DOI:** 10.3389/fbioe.2020.00989

**Published:** 2020-08-13

**Authors:** Rocio J. Jimenez-Valdes, Uryan I. Can, Brian F. Niemeyer, Kambez H. Benam

**Affiliations:** ^1^Division of Pulmonary Sciences and Critical Care Medicine, Department of Medicine, University of Colorado Anschutz Medical Campus, Aurora, CO, United States; ^2^Department of Bioengineering, University of Colorado Denver, Aurora, CO, United States; ^3^Linda Crnic Institute for Down Syndrome, University of Colorado Anschutz Medical Campus, Aurora, CO, United States

**Keywords:** spheroids, organoids, bio-scaffolds, precision cut lung slices, bronchial biopsies, 3D bioprinting, lung-on-a-chip, inhalation models

## Abstract

Lung disorders such as chronic obstructive pulmonary disease (COPD) and lower respiratory tract infections (LRTIs) are leading causes of death in humans globally. Cigarette smoking is the principal risk factor for the development of COPD, and LRTIs are caused by inhaling respiratory pathogens. Thus, a thorough understanding of host–environment/pathogen interactions is crucial to developing effective preventive and therapeutic modalities against these disorders. While animal models of human pulmonary conditions have been widely utilized, they suffer major drawbacks due to inter-species differences, hindering clinical translation. Here we summarize recent advances in generating complex 3D culture systems that emulate the microarchitecture and pathophysiology of the human lung, and how these platforms have been implemented for studying exposure to environmental factors, airborne pathogens, and therapeutic agents.

## Introduction

Lung disorders represent a great socioeconomic challenge and a major burden on health care systems worldwide; chronic obstructive pulmonary disease (COPD) and lower respiratory tract infections (LRTIs) are the third and fourth leading causes of death globally, respectively (World Health Organization [WHO], 2018). Cigarette smoke exposure is the principal risk factor for COPD development and exacerbation, and LRTIs are consequent to inhalation of airborne pathogens. Therefore, a better understanding of the pulmonary system at health and disease and recreation of respiratory host–environment/pathogen interactions are crucial if we are to prevent and treat lung disorders. While animal models of human respiratory conditions have been instrumental in advancing our knowledge, they suffer major drawbacks due to inter-species (e.g., genetic, homeostatic physiology, pathology, and respiratory tree anatomy, airway histology, etc.) differences hindering clinical translation. Rodent animal models, for instance, which have widely been applied to shed light on signaling networks and lung development, are fundamentally different from humans in terms of lung cellular composition, airway branching, and immune system function (Rackley and Stripp, 2012). Besides, rodents are obligate nose breathers with intricate and highly developed nasal turbinates that lead to different particulate deposition patterns compared to humans during breathing (Hecht, 2005). Due to these factors, rodents at best are passive inhalers and, unlike humans, are unable to actively inhale therapeutic candidates or cigarette smoke.

To circumvent some of these shortcomings with animal models, simple two-dimensional (2D) cell culture models that emulate naturally present lung air–liquid interface (ALI) have been developed to study human lung pathophysiology *in vitro* (Prunieras et al., 1983). Recently, these systems have been used in combination with cell exposure systems that mimic the natural route of exposure of lung epithelium to environmental factors in order to measure the risks of potentially damaging inhaled nanoparticles (Lenz et al., 2009) or cigarette smoke (Thorne and Adamson, 2013). However, despite their use of primary human lung airway epithelial cells (hAECs) and recapitulation of mucociliated histology, they have several limitations in recreating organ-level functionalities of the human lung. For example, they do not enable (1) real-time analysis of dynamic intercellular (e.g., leukocyte-endothelial cell) interactions under physiological flow, (2) study of inhalation exposure to whole cigarette smoke, as a representative inhaled material, under physiological breathing airflow without disturbing ALI, (3) recreation of blood-like vascular perfusion to continually supply nutrients and growth factors, (4) introduction and perturbation of vascular and airflow shear stress to simulate different pathophysiological conditions, and (5) high-resolution kinetic analysis of biological responses (e.g., the time-course release of secreted factors, like cytokines/chemokines, in response to pathogenic challenge) (Fang and Eglen, 2017; Ainslie et al., 2019). In addition, these platforms are commonly used in the absence of sub-epithelial extracellular matrix (ECM; Yamaya et al., 1992; Gray et al., 1996).

Recent advances in tissue engineering and microfabrication have led to the development and application of more complex 3D culture systems and biomimetic microfluidic platforms to capture the structural and functional complexity of the human lung (Miller and Spence, 2017). In this review, we assess these state-of-the-art complex *in vitro* lung models, and the efforts made to expose these models to environmental factors, pathogens, and therapeutic agents. Finally, we discuss future directions on tackling the challenges of *in vitro* lung models that were observed over the past decade and share our vision on how to further enhance these models for more accurate and better clinical translation.

## Recent Advances in Creating Complex 3D *in vitro* Models of Human Lung

### Spheroids and Organoids

Among the simplest of the 3D lung models are spheroids. Spheroids are multicellular sphere-like culture systems that enable recapitulation of cell–cell interactions; they are either utilized as freely floating cell aggregates or get seeded onto 3D bio-scaffolds (Ainslie et al., 2019). Spheroids utilize tumor-derived cells, patient-derived xenograft cells, and immortalized cell lines, and have been applied to study intercellular interactions and response to therapies in the context of cancer (Ekert et al., 2014; Meenach et al., 2016; Klameth et al., 2017; Lewis et al., 2018), lung progenitor cells differentiation (Chimenti et al., 2017), and pulmonary fibrosis (Surolia et al., 2017). However, spheroid culture systems still suffer deficiencies such as a lack of vasculature and ALI, hard to control cell ratios and aggregate size, as well as a failure to mimic organ function (Fang and Eglen, 2017); therefore, spheroids do not represent good *in vitro* lung models to study host–environment/pathogen interactions. Another similar yet distinct preclinical system is organoids; these are cultured organ-specific cell types, which are derived from a population of stem cells (adult or pluripotent), and are capable of maintaining stem cells during *in vitro* culture. During formation, organoids develop into 3D tissues that recreate *in vivo*-observed microanatomy through self-organization (Fang and Eglen, 2017; Ainslie et al., 2019). Compared to spheroids, organoids exhibit long-term viability and rely on internal developmental processes (rather than cell–cell adhesions) to drive tissue/organ-like microarchitecture formation. Human lung organoids have been generated to replicate bronchi/bronchioles (Konishi et al., 2016; Mccauley et al., 2017; Miller et al., 2018), alveoli (Zacharias et al., 2018), and even multi-lineage structures (Dye et al., 2016). While helpful in advancing our understanding of the respiratory system, particularly from a developmental perspective, this models lack several of key organ features such as vascularization, and mechanical forces associated with breathing, and it is difficult to obtain fully differentiated lung cell types (Barkauskas et al., 2017). Lung organoids have been sparsely used for host–environment/pathogen exposure studies, since the organoid lumen, which represents the apical surface of the lungs through which a natural exposure would occur *in vivo*, is difficult to access, thus treatments or stimuli are applied in the environment in which the organoids are embedded. In this scenario, murine organoids have been infected with influenza virus (Quantius et al., 2016), and human organoids have been infected with respiratory syncytial virus (RSV; Sachs et al., 2019). Some attempts have been made to microinject pathogens or their by-products into organoids using custom made tools, primarily in gastrointestinal organoids (Williamson et al., 2018); however, in lung organoids, this technique has only been applied to propagate Cryptosporidium, a protozoan parasite (Heo et al., 2018).

### Precision Cut Lung Slices

To address the disconnection that often exists in spheroids and organoids in including both cellular and organ-level complexities *in vitro*, some groups have turned to an *ex vivo* system called precision cut lung slices (PCLS), which maintain the cellular structure and the biological processes of the lung (Ainslie et al., 2019). Importantly, PCLS generation from healthy explants and diseased tissue can reveal differences in the cellular and molecular interactions within the microenvironment of the lung. Disease modeling of healthy tissue can be achieved *ex vivo* through mimicking disease characteristics and used for exploring therapeutic treatments (Alsafadi et al., 2020). Precision cut lung slices have been applied for cytotoxicity assessment to low-molecular-weight (LMW) chemicals (Lauenstein et al., 2014), evaluation of biological responses to inflammatory stimuli such as lipopolysaccharide endotoxin (LPS), macrophage-activating lipopeptide-2 (MALP-2), interferon gamma (IFNgamma) (Henjakovic et al., 2008), and recreation of pathologies such as pulmonary fibrosis, through the use of a combination of profibrotic growth factors and signaling molecules (Alsafadi et al., 2017) and COPD models induced with 1-(3-Hydroxy-5-(thiophen-2-yl)phenyl)-3-(naphthalen-2-yl)urea (FzM1) (Skronska-Wasek et al., 2017). However, PCLS are often cultivated only for short periods of time (they are very challenging to maintain viable and functional for weeks), their use is impacted by the limited availability of whole/resected organ for slicing, and they only represent a brief snapshot of the cell populations in the tissue. Besides, the PCLS cannot fully recapitulate all attributes seen *in vivo*; for instance, there are major challenges with preserving arteriole morphology and localization of lung airway smooth muscle cells (Sanderson, 2011). Precision cut lung slices can reproduce the initial interactions and inflammatory responses to industrial chemicals (Lauenstein et al., 2014) and pathogens [e.g., influenza viruses (Liu et al., 2015), rhinovirus (Beale et al., 2014), *Yersinia pestis* (Banerjee et al., 2019), and *Coxiella burnetiid* (Graham et al., 2016)] in the human respiratory system; however, the extent to which the immune response can replicate the *in vivo* situation is limited, as demonstrated by Neuhaus et al. (2017), who observed that LPS-induced tumor necrosis factor-alpha (TNF-α) secretion decreased significantly over a period of 15 days, and it is not possible to recruit non-resident immune cells. Moreover, while murine-derived PCLS models have been adapted to study the effects of cigarette smoke (Donovan et al., 2016), the PCLS per se have not been utilized for inhalation exposure studies *in vitro*, principally because the route of administration represents a challenge (e.g., physiological ALI cannot be established in this model system), and often the entire slice is bathed in the compound or stimulant of interest (Liu et al., 2019). Changing from ALI conditions to liquid-submerged conditions represents a drawback, as it impacts epithelial cells’ glycoprotein secretion profile, tight junction integrity, and permeability (Grainger et al., 2006). In addition, the physio-chemical properties of pulmonary stimuli such as airborne pollutants, gaseous substances, novel medications, and therapeutic agents can alter when in liquid suspension (Upadhyay and Palmberg, 2018). In addition to this, PCLS become static systems, lacking the shear flows associated with air and blood flow in the ALI and vascular endothelium, respectively, which could impact cell–cell/ECM responses and interactions in tissues.

### Bronchial Biopses

Bronchial biopsies are samples of airway tissue obtained from the carinae of large and small cartilaginous airways. These samples are often fixed and used to measure airway remodeling by morphometric analyses of airway epithelial mucin stores, measurements of reticular basement membrane thickness, quantification of the number and size of globlet cells, the analysis of the content and density of the smooth muscle of the airways, as well as quantification and characterization of inflammatory cells (Woodruff and Innes, 2006). In addition, some effort have been made to culture the bronchial biopsies for short-term exposure studies *in vitro*. For instance, this culture model has been used to study the effects of chemotherapeutic treatments on non-small cell lung cancer (Lang et al., 2007), to evaluate cellular responses by exposing asthmatic lung tissue to allergen *Dermatophagoides pteronyssinus* (Jaffar et al., 1999; Lordan et al., 2001; Vijayanand et al., 2010), and to test the antiviral efficacy of therapeutic drugs against influenza virus infection (Nicholas et al., 2015). The advantages of bronchial biopsies include retaining the three-dimensional structure of the lungs if the extraction procedure is successful, which requires a trained bronchoscopist, allowing sampling from healthy subjects as well as patients with lung diseases, and the minimally invasive nature of the sampling procedure. However, this system has similar drawbacks as the PCLS, and is impacted by scarcity of donor tissue availability, small sample size (usually 1–3 mm) and number of biopses that can be obtained at any given time (∼4–10). In addition the *in vitro* viability in often low, therefore exposure studies on bronchial biopsies usually do not last more than a single day.

### Cellularized Bio-Scaffolds

Extracellular matrix is the 3D network of extracellular macromolecules that provide structural integrity and biochemical support to tissue-resident cells. Extracellular matrix both serves as a scaffold and modulates cellular responses such as self-renewal, quiescence, migration, proliferation, phenotype maintenance, differentiation, and apoptosis (Akhmanova et al., 2015). Hydrogels can mimic ECM *in vitro* using natural products (like collagen, hyaluronic acid, chitosan, alginate, or Matrigel) or synthetic polymers [such as polyethylene glycol (PEG) or polyacrylamide (PAA)] (Zhou et al., 2018). Natural hydrogels have been predominantly used to culture lung cells and organoids (Sato et al., 2017; Zacharias et al., 2018); however, artificial matrices have been demonstrated as a viable alternative (Miller et al., 2019), since their components are biologically inert, and can be functionalized by the addition of proteins, peptides, and/or polysaccharides (Akhmanova et al., 2015).

Besides hydrogels, lung ECM can be generated via decellularization of the whole/resected lung through perfusion with detergents, which allows considerable preservation of the micro- and macro-architecture of the organ, and the ECM composition (Gilpin and Wagner, 2018). Extracellular matrix has been recognized as a bioactive medium that modulates cellular responses in its surroundings. By using cellularized bio-scaffolds it will therefore be possible to mimic some pathological alterations, and the consequent functional changes that occur in lung diseases, especially those with a chronic nature (Burgess et al., 2016). Decellularized lung scaffolds have enabled studying cell–ECM interactions in tissues of healthy people and patients with chronic lung diseases, such as lung cancer (Stratmann et al., 2014), idiopathic pulmonary fibrosis (IPF; Van Der Velden et al., 2018), and scleroderma (Sun et al., 2016). These matrices are instrumental in evaluating lung repair and regeneration and have been recellularized with different types of human cells, including fibroblasts, endothelial cells, epithelial cells, stem cells from multiple organ sources, and organoids (Porzionato et al., 2018; Giobbe et al., 2019). However, it has not been possible to completely recellularize these matrices to allow recreation of lung physiology and function, their application has been partly hindered due to the limited number of whole/resected human lungs, and to our knowledge, their utilization [beyond regenerative medicine and orthotopic transplantation (Guenthart et al., 2019)] for studying host–environment/pathogen interactions has not been demonstrated.

### 3D Bioprinting

To more faithfully replicate tissue microarchitectures of the human lung, 3D bioprinting has emerged as a great technological platform ideally positioned to create finely defined and controlled biological structures and living systems by utilizing a wide array of materials (natural, synthetic, or even hybrid hydrogels) and cell types. This technology can achieve a high degree of precision in cell positioning and can be scalable for automation (Gungor-Ozkerim et al., 2018). However, 3D bioprinting has been predominantly applied for the development of vascularized tissues (Kolesky et al., 2014), rather than generating functional whole organ or organ-like mimicries. To our knowledge, only a few studies have focused on the lungs. Horvath et al. (2015) biofabricated a three-part mimetic (composed of an endothelial cell layer, basement membrane, and an epithelial cell layer) of air–blood barrier analog of the human lung alveoli within a transwell insert. While a great initiative, the study utilized cell lines, rather than primary human-derived cells, and the authors only focused on model validation (viability, cellular proliferation, and barrier function) and no data were presented on the application of the platform for the analysis of host-environment/pathogen interactions. More recently, a new method for 3D bioprinting of tissues by stereolithography was reported, whereby it was possible to make a model inspired by alveolar morphology, capable of withstanding mechanical strain for cyclical ventilation and oxygen transport (Grigoryan et al., 2019). While successful in creating entangled vascular networks of the lung alveoli, the bioprinted tissue lacked alveolar epithelial cells, and the platform was applied neither for toxicological studies nor for inhaled exposure to a respiratory pathogen or therapeutic agents. Altogether, 3D bioprinting offers the possibility of developing biologically inspired lung organotypic models; however, despite the advantages of 3D bioprinting, wide spread utilization of these techniques has been limited by the requirement of expensive and complex technologies as well as a steep learning curve.

### Lung-on-a-Chip Models

Organ-on-a-Chips are biomimetic, microfluidic, cell culture devices created with microchip manufacturing methods that contain continuously perfused hollow microchannels inhabited by living tissue cells arranged to simulate organ-level physiology (Bhatia and Ingber, 2014; Benam et al., 2015). By recapitulating the multicellular architectures, tissue–tissue interfaces, chemical gradients, mechanical cues, and vascular perfusion of the body, these devices produce levels of tissue and organ functionality with well-defined structures and highly controlled microenvironments that would not be possible with conventional 2D or 3D culture systems. They also enable high-resolution, real-time imaging and *in vitro* analysis of biochemical, genetic and metabolic activities of living human cells in a functional human tissue and organ context. Adaptation of Organ-on-a-Chip technology by pulmonary scientists has led to the development of model systems that emulate human lung pathophysiology *in vitro*.

Lung-on-a-Chip devices have been developed to model different regions of the respiratory system and recapitulate *in vivo*-observed multicellular architecture and physicochemical environment of the lung (airway and alveoli). The use of this technology (Alveolus-on-a-Chip) has enabled a better understanding of different aspects of human alveolar pathologies; such as evaluating the influence of breathing-associated mechanical cues on the growth and migration pattern of Non-Small Cell Lung Cancer tumor cells (NSCLC; Hassell et al., 2017), reproducing interleukin-2 (IL-2) induced alveolar edema observed in human cancer patients at similar doses and over the same time frame, and its pharmacological inhibition (Huh et al., 2012), mimicking pulmonary thrombosis by treatment with inflammatory stimuli (TNF-α) and bacterial products (LPS) corroborated in murine models (Jain et al., 2018), studying inhaled exposure of the alveolar epithelium to aerosolized LPS and its immune-modulatory impact (Artzy-Schnirman et al., 2019), and recreating recruitment of circulating leukocytes and inflammation induction after treatment of epithelial cells with TNF-a, *Escherichia coli*, and silica nanoparticles (Huh et al., 2010). In these microphysiological systems primary, cancerous or immortalized cell line of human alveolar epithelial origin have been co-cultured with human lung microvascular endothelial cells (Huh et al., 2010, 2012; Hassell et al., 2017) or human umbilical vascular endothelial cells (HUVECs; Jain et al., 2018).

In the context of conducting airways, we have developed and applied Lung-on-a-Chip devices (Airway-on-a-Chip) for culture and differentiation of primary human epithelial cells in co-culture with primary human lung microvascular endothelial cells, to model small airways, reproduce the mechanical forces associated with inhalation-exhalation respiration cycles (airflow shear), mimic the shear forces across endothelial cell layers (via vascular flow), and recreate the ALI barrier of human airways (Benam et al., 2015,2016a,b,2017,2018,2019,2015; Benam and Ingber, 2016; Niemeyer et al., 2018). This technology has been adapted to allow first-in-kind studies that reproduce and characterize tissue–tissue crosstalk between pulmonary epithelium and airway smooth muscle (Humayun et al., 2018) and enable better understanding infectious disease biogenesis processes through analysis of the production of inflammatory cytokines and immune cell recruitment (neutrophils) following exposure to respiratory fungi (e.g., *Aspergillus fumigatus*) and the bacteria (e.g., *Pseudomonas aeruginosa*) (Barkal et al., 2017) by other investigators. Similay utilizing Airway-on-a-Chip we have been able to dissect inflammatory processes involved in the pathobiology of chronic lung conditions such as asthma induced by the exposure of ephitelium to IL-13 and COPD by recreating terminally differentiated mucociliated airway epithelia on-chip, and exposing cells to the viral mimic polyinosinic–polycytidylic acid (poly(I:C)) or to LPS (Benam et al., 2016b), and identify novel biomarkers of human lung diseases and test efficacy of lead therapeutic compounds (Benam et al., 2016b).

We developed a Breathing-Smoking Lung-on-a-Chip platform that recreates smoke-induced pathologies in humans (Benam et al., 2016b). The system consisted of a Lung Small Airway-on-a-Chip (Benam et al., 2016b) that reproduces the living bronchiolar tissue for exposure to inhaled whole cigarette smoke (WCS), a “microrespirator” that emulates diaphragm and rib cage function and reproduces inhalation-exhalation airflow at physiological rhythms and patterns, a “biomimetic smoking robot” (Benam et al., 2020) that mimics human mouth and generates fresh WCS and regulates the passage of inhale/exhale air/smoke, and software that represents the brain of human smoker and controls “smoking topography” and “breathing” behavior. Using this platform, we flowed WCS horizontally across the apical surface of differentiated human bronchiolar epithelia (as occurs in the lungs of human smokers), from normal subjects and COPD patients, at representative smoking topography parameters for puff time, puff volume and inter-puff interval under clinically relevant breathing conditions. We found that inhaled smoke exposure transformed ciliary beat frequency (CBF) pattern, range, and variability and induced oxidative stress. Importantly, it was observed that submerging the ciliated airway epithelia with cell culture medium alone was sufficient to mask the impact of inhaled WCS on ciliary micro-pathologies. In addition, we discovered new smoke-mounted biological markers that distinguish COPD epithelia from those from healthy individuals (Benam et al., 2016a).

Recently, Elias-Kirma et al. (2020) developed an interesting model of branching Airway-on-a-Chip for toxicological evaluation of inhaled particulate matter. The authors utilized primary human bronchial epithelial cells for culture, and exposed the epithelium on-chip to anti-vanilloid receptor 1 (VR1) antibody-coated polystyrene microparticles. The microparticles were generated using a commercial aerosolizing machine and brought into contact with the cells through a pinch valve-controlled antistatic tube. While the device design in this study was unique, the exposure did not mimic what naturally occurs during rhythmic breathing cycles and the authors did not study biological impact of exposure to environmental pollutants, tobacco products or therapeutic agents.

These study implies that Lung-on-a-Chip platforms have the potential for recreation of *in vivo*-like inhaled exposure to pathogens, environmental threats, and drug treatments. However, for such platforms to be more applicable and physiologically relevant, it is important to enhance their multicellularity and complexity, for instance by the addition of parenchymal cells, such as fibroblasts, tissue-resident and circulating immune cells, muscle cells, and/or ECM. One of the drawbacks of microfluidics is that manufacturing is often expensive and time consuming; in addition, instruments such as syringe pumps or air pressure systems are required to control fluid flow within the chips. Furthermore, the devices need to become more user friendly (particularly for biologists with little/no engineering expertise) and be amenable for increased throughput. On the other hand, although microphysiological systems have great flexibility with respect to the experimental design, there are important challenges due to miniaturization, such as reduced media volumes, different media exchange rates and methods, and lower number of cells tested; finally, and an additional challenge in conducting host-pathogen/environment studies is the stimulus dose. Therefore, it is necessary to carefully evaluate these differences when comparing cell behavior and viability in microfluidic devices versus macroscopic cultures, and especially when comparing against *in vivo* results to validate the systems. Moreover, from a high-level perspective investigators utilizing Organ-on-Chip devices must consider as accurate and as feaible as possible validating and qualifying these models against tissues that they are aiming to replicate – i.e., human samples and clinical data. Lastly, we would like to mention that multiple factors have contributed to popularity and quick and widespread adaptation of PDMS for fabrication of Lung-on-a-Chip microfluidic devices. These include ability for rapid prototyping and multilayer device fabrication, optical transparency from 240 to 1100 nm (enabling utilization by various optical detection schemes), flexibility (allowing axial stretch), gas-permeablity (so that oxygen can feasibly penetrate the device and reach the cells embedded within the chip), being non-toxic, inexpensive and not breaking (like glass). However, a drawback of PDMS has been its absorption of small, hydrophobic molecules from flowing solution (Toepke and Beebe, 2006). To mitigate this, we apply either of these two approaches: (1) correcting for absorption of small hydrophobic molecules by quantifying the loss (e.g., via mass spectrometry) and (2) saturating the chips prior to cell culture or experimentation to minimize loss.

## Conclusion and Future Directions

**FIGURE 1:**
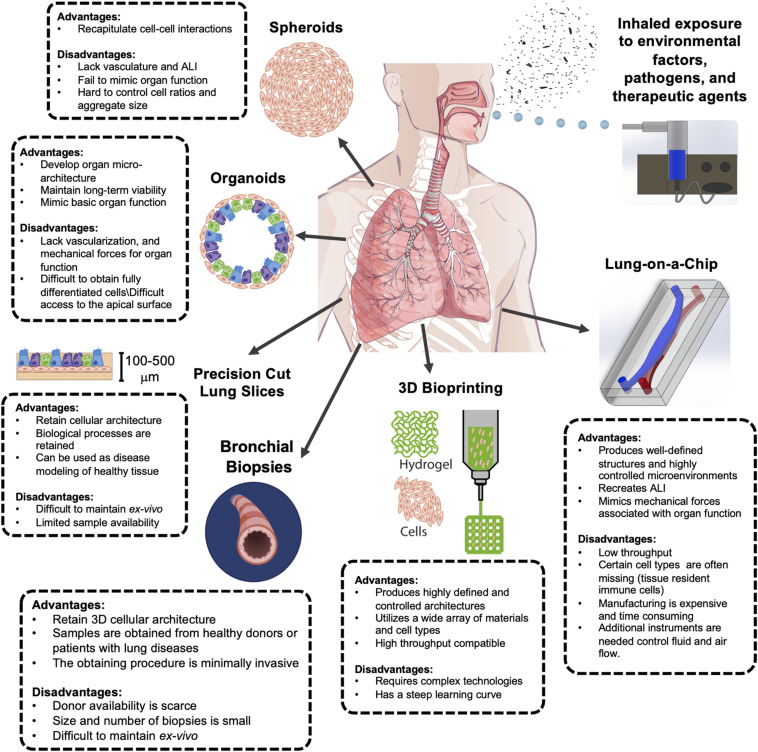
Advantages and disadvantages of complex 3D *in vitro* culture models of the human lung for inhalation exposure studies. Each model presented has unique benefits and challenges. Improved airway modeling can be achieved through incorporating inhalation exposure systems. The digital images of human lung at the center and bronchus near the bottom left were acquired from Shutterstock and Biorender, respectively.

In this article, we reviewed the most recent advances in creating preclinical 3D model systems that reproduce human lung pathophysiology *in vitro* and discussed their respective merits and drawbacks in the context of host-environment/pathogen interactions (Figure 1). It has been a long road for the transition from 2D to 3D cell culture systems; however, 3D models are gaining popularity in use and adaptation due to their critical advantages over static cell cultures. Spheroids, organoids, and PCLS have proven to be invaluable tools for studying lung development and pathologies, but they lack natural ALI and are unable to reproduce breathing-associated airflow, thus it is not possible to perform inhalation exposure studies using these platforms. Cellularized scaffolds have been widely used to study cell–ECM interactions at health and disease, and for regenerative purposes; however, these have not yet been used to assess host–environment/pathogen interactions. Recently, 3D bioprinting has emerged as a promising technique for developing more intricate lung living systems with great potential for high throughput yield; but numerous technical challenges need to be tackled and complex-system building to integrate multiple organ-specific cells and tissue (beyond generating vascularized constructs) must become a priority. In contrast, microfluidic Lung-on-a-Chip microdevices have emerged as an attractive alternative to mimic lung function and biology as seen *in vivo*. In the particular case of lung models, and more specifically in the study of the host–environment/pathogen interaction, it is important how the treatments and/or stimuli are delivered, in this sense, the microfluidic platforms have demonstrated to be very robust and physiologically relevant *in vitro* tools that can incorporate biochemical, physical and mechanical cues. We envision considerable improvement and adaption of Lung-on-a-Chip platforms for the study of host–environment/pathogen interactions. The improvements include allowing higher throughput analysis, and integration of ECM and additional cellular complexity. These microdevices will be utilized for the study of nebulized and dry powder treatments and recreation of clinically relevant exposure to airborne respiratory pathogens (instead of lung cells being exposed submerged to the infective agents). We also anticipate the *in vitro* cell culture of cellularized matrices and potentially PCLS within the Lung-on-a-Chip system to provide dynamism and enhanced viability *ex vivo*. In line with this, 3D bioprinting techniques could revolutionize the way microfluidic devices are currently manufactured, allowing for increased production and reproducibility.

## Author Contributions

KB conceptualized and critically revised the manuscript. RJ-V, UC, and BN drafted the manuscript. All authors contributed to the article and approved the submitted version.

## Conflict of Interest

KB is the founder and holds equity in Pneumax, LLC. KB and BN are co-inventors on patent applications describing Lung Chip and related technologies. The remaining authors declare that the research was conducted in the absence of any commercial or financial relationships that could be construed as a potential conflict of interest.

## References

[B1] Ainslie G. R. Davis M. Ewart L. Lieberman L. A. Rowlands D. J. Thorley A. J. (2019). Microphysiological lung models to evaluate the safety of new pharmaceutical modalities: a biopharmaceutical perspective. *Lab Chip* 19 3152–3161. 31469131 10.1039/c9lc00492k

[B2] Akhmanova M. Osidak E. Domogatsky S. Rodin S. Domogatskaya A. (2015). Physical, spatial, and molecular aspects of extracellular matrix of *in vivo* niches and artificial scaffolds relevant to stem cells research. *Stem Cells Int.* 2015:167025. 10.1155/2015/167025 PMC4553184 26351461

[B3] Alsafadi H. N. Staab-Weijnitz C. A. Lehmann M. Lindner M. Peschel B. Konigshoff M. (2017). An *ex vivo* model to induce early fibrosis-like changes in human precision-cut lung slices. *Am. J. Physiol. Lung Cell. Mol. Physiol.* 312 L896–L902. 28314802 10.1152/ajplung.00084.2017

[B4] Alsafadi H. N. Uhl F. E. Pineda R. H. Bailey K. E. Rojas M. Wagner D. E. (2020). Applications and approaches for three-dimensional precision-cut lung slices. Disease modeling and drug discovery. *Am. J. Respir. Cell Mol. Biol.* 62 681–691. 10.1165/rcmb.2019-0276tr 31991090 PMC7401444

[B5] Artzy-Schnirman A. Zidan H. Elias-Kirma S. Ben-Porat L. Tenenbaum-Katan J. Carius P. (2019). Capturing the onset of bacterial pulmonary infection in acini-on-chips. *Advan. Biosys.* 3:e1900026. 10.1002/adbi.201900026 PMC7611792 32648651

[B6] Banerjee S. K. Huckuntod S. D. Mills S. D. Kurten R. C. Pechous R. D. (2019). Modeling pneumonic plague in human precision-cut lung slices highlights a role for the plasminogen activator protease in facilitating type 3 secretion. *Infect. Immun.* 87:e00175-19. 10.1128/IAI.00175-19 PMC6652753 31085709

[B7] Barkal L. J. Procknow C. L. Alvarez-Garcia Y. R. Niu M. Jimenez-Torres J. A. Brockman-Schneider R. A. (2017). Microbial volatile communication in human organotypic lung models. *Nat. Commun.* 8:1770. 10.1038/s41467-017-01985-4 PMC5701243 29176665

[B8] Barkauskas C. E. Chung M. I. Fioret B. Gao X. Katsura H. Hogan B. L. (2017). Lung organoids: current uses and future promise. *Development* 144 986–997. 10.1242/dev.140103 28292845 PMC5358104

[B9] Beale J. Jayaraman A. Jackson D. J. Macintyre J. D. R. Edwards M. R. Walton R. P. (2014). Rhinovirus-induced IL-25 in asthma exacerbation drives type 2 immunity and allergic pulmonary inflammation. *Sci. Transl. Med.* 6:256ra134. 10.1126/scitranslmed.3009124 25273095 PMC4246061

[B10] Benam K. H. Dauth S. Hassell B. Herland A. Jain A. Jang K. J. (2015). Engineered in vitro disease models. *Annu. Rev. Pathol.* 10 195–262. 25621660 10.1146/annurev-pathol-012414-040418

[B11] Benam K. H. Gilchrist S. Kleensang A. Satz A. B. Willett C. Zhang Q. (2019). Exploring new technologies in biomedical research. *Drug Discov. Today* 24 1242–1247. 10.1016/j.drudis.2019.04.001 30953865

[B12] Benam K. H. Ingber D. E. (2016). Commendation for exposing key advantage of organ chip approach. *Cell Syst.* 3:411. 10.1016/j.cels.2016.11.009 27883885

[B13] Benam K. H. Konigshoff M. Eickelberg O. (2018). Breaking the in vitro barrier in respiratory medicine. engineered microphysiological systems for chronic obstructive pulmonary disease and beyond. *Am. J. Respir. Crit. Care Med.* 197 869–875. 10.1164/rccm.201709-1795pp 29262260

[B14] Benam K. H. Mazur M. Choe Y. Ferrante T. C. Novak R. Ingber D. E. (2017). Human lung small airway-on-a-chip protocol. *Methods Mol. Biol.* 1612 345–365. 10.1007/978-1-4939-7021-6_25 28634955

[B15] Benam K. H. Novak R. Ferrante T. C. Choe Y. Ingber D. E. (2020). Biomimetic smoking robot for in vitro inhalation exposure compatible with microfluidic organ chips. *Nat. Protoc.* 15 183–206. 10.1038/s41596-019-0230-y 31925401

[B16] Benam K. H. Novak R. Nawroth J. Hirano-Kobayashi M. Ferrante T. C. Choe Y. (2016a). Matched-comparative modeling of normal and diseased human airway responses using a microengineered breathing lung chip. *Cell Syst.* 3 456–466.e4. 10.1016/j.cels.2016.10.003 27894999

[B17] Benam K. H. Villenave R. Lucchesi C. Varone A. Hubeau C. Lee H. H. (2016b). Small airway-on-a-chip enables analysis of human lung inflammation and drug responses *in vitro*. *Nat. Methods* 13 151–157. 10.1038/nmeth.3697 26689262

[B18] Bhatia S. N. Ingber D. E. (2014). Microfluidic organs-on-chips. *Nat. Biotechnol.* 32 760–772. 10.1038/nbt.2989 25093883

[B19] Burgess J. K. Mauad T. Tjin G. Karlsson J. C. Westergren-Thorsson G. (2016). The extracellular matrix - the under-recognized element in lung disease? *J. Pathol.* 240 397–409. 10.1002/path.4808 27623753 PMC5129494

[B20] Chimenti I. Pagano F. Angelini F. Siciliano C. Mangino G. Picchio V. (2017). Human lung spheroids as *in vitro* niches of lung progenitor cells with distinctive paracrine and plasticity properties. *Stem Cells Transl. Med.* 6 767–777. 10.5966/sctm.2015-0374 28297570 PMC5442776

[B21] Donovan C. Seow H. J. Bourke J. E. Vlahos R. (2016). Influenza A virus infection and cigarette smoke impair bronchodilator responsiveness to beta-adrenoceptor agonists in mouse lung. *Clin. Sci.* 130 829–837. 10.1042/cs20160093 27128803 PMC5233570

[B22] Dye B. R. Dedhia P. H. Miller A. J. Nagy M. S. White E. S. Shea L. D. (2016). A bioengineered niche promotes *in vivo* engraftment and maturation of pluripotent stem cell derived human lung organoids. *eLife* 5:e19732. 10.7554/eLife.19732 PMC5089859 27677847

[B23] Ekert J. E. Johnson K. Strake B. Pardinas J. Jarantow S. Perkinson R. (2014). Three-dimensional lung tumor microenvironment modulates therapeutic compound responsiveness *in vitro*–implication for drug development. *PLoS One* 9:e92248. 10.1371/journal.pone.0092248 24638075 PMC3956916

[B24] Elias-Kirma S. Artzy-Schnirman A. Das P. Heller-Algazi M. Korin N. Sznitman J. (2020). *In situ*-like aerosol inhalation exposure for cytotoxicity assessment using airway-on-chips platforms. *Front. Bioeng. Biotechnol.* 8:91. 10.3389/fbioe.2020.00091 32154228 PMC7044134

[B25] Fang Y. Eglen R. M. (2017). Three-dimensional cell cultures in drug discovery and development. *SLAS Discov.* 22 456–472. 10.1177/1087057117696795 28520521 PMC5448717

[B26] Gilpin S. E. Wagner D. E. (2018). Acellular human lung scaffolds to model lung disease and tissue regeneration. *Eur. Respir. Rev.* 27:180021. 10.1183/16000617.0021-2018 29875137 PMC9488127

[B27] Giobbe G. G. Crowley C. Luni C. Campinoti S. Khedr M. Kretzschmar K. (2019). Extracellular matrix hydrogel derived from decellularized tissues enables endodermal organoid culture. *Nat. Commun.* 10:5658. 10.1038/s41467-019-13605-4 PMC6906306 31827102

[B28] Graham J. G. Winchell C. G. Kurten R. C. Voth D. E. (2016). Development of an *ex vivo* tissue platform to study the human lung response to *Coxiella burnetii*. *Infect. Immun.* 84 1438–1445. 10.1128/iai.00012-16 26902725 PMC4862715

[B29] Grainger C. I. Greenwell L. L. Lockley D. J. Martin G. P. Forbes B. (2006). Culture of Calu-3 cells at the air interface provides a representative model of the airway epithelial barrier. *Pharm. Res.* 23 1482–1490. 10.1007/s11095-006-0255-0 16779708

[B30] Gray T. E. Guzman K. Davis C. W. Abdullah L. H. Nettesheim P. (1996). Mucociliary differentiation of serially passaged normal human tracheobronchial epithelial cells. *Am. J. Respir. Cell Mol. Biol.* 14 104–112. 10.1165/ajrcmb.14.1.8534481 8534481

[B31] Grigoryan B. Paulsen S. J. Corbett D. C. Sazer D. W. Fortin C. L. Zaita A. J. (2019). Multivascular networks and functional intravascular topologies within biocompatible hydrogels. *Science* 364 458–464. 10.1126/science.aav9750 31048486 PMC7769170

[B32] Guenthart B. A. O’neill J. D. Kim J. Fung K. Vunjak-Novakovic G. Bacchetta M. (2019). Cell replacement in human lung bioengineering. *J. Heart Lung Transplant.* 38 215–224. 10.1016/j.healun.2018.11.007 30529200 PMC6351169

[B33] Gungor-Ozkerim P. S. Inci I. Zhang Y. S. Khademhosseini A. Dokmeci M. R. (2018). Bioinks for 3D bioprinting: an overview. *Biomater. Sci.* 6 915–946. 29492503 10.1039/c7bm00765e PMC6439477

[B34] Hassell B. A. Goyal G. Lee E. Sontheimer-Phelps A. Levy O. Chen C. S. (2017). Human organ chip models recapitulate orthotopic lung cancer growth, therapeutic responses, and tumor dormancy *in vitro*. *Cell Rep.* 21 508–516. 10.1016/j.celrep.2017.09.043 29020635

[B35] Hecht S. S. (2005). Carcinogenicity studies of inhaled cigarette smoke in laboratory animals: old and new. *Carcinogenesis* 26 1488–1492. 10.1093/carcin/bgi148 15930027

[B36] Henjakovic M. Sewald K. Switalla S. Kaiser D. Muller M. Veres T. Z. (2008). *Ex vivo* testing of immune responses in precision-cut lung slices. *Toxicol. Appl. Pharmacol.* 231 68–76. 10.1016/j.taap.2008.04.003 18504053

[B37] Heo I. Dutta D. Schaefer D. A. Iakobachvili N. Artegiani B. Sachs N. (2018). Modelling cryptosporidium infection in human small intestinal and lung organoids. *Nat. Microbiol.* 3 814–823. 10.1038/s41564-018-0177-8 29946163 PMC6027984

[B38] Horvath L. Umehara Y. Jud C. Blank F. Petri-Fink A. Rothen-Rutishauser B. (2015). Engineering an in vitro air-blood barrier by 3D bioprinting. *Sci. Rep.* 5:7974. 10.1038/srep07974 PMC4303938 25609567

[B39] Huh D. Leslie D. C. Matthews B. D. Fraser J. P. Jurek S. Hamilton G. A. (2012). A human disease model of drug toxicity-induced pulmonary edema in a lung-on-a-chip microdevice. *Sci. Transl. Med.* 4:159ra147. 10.1126/scitranslmed.3004249 23136042 PMC8265389

[B40] Huh D. Matthews B. D. Mammoto A. Montoya-Zavala M. Hsin H. Y. Ingber D. E. (2010). Reconstituting organ-level lung functions on a chip. *Science* 328 1662–1668. 10.1126/science.1188302 20576885 PMC8335790

[B41] Humayun M. Chow C. W. Young E. W. K. (2018). Microfluidic lung airway-on-a-chip with arrayable suspended gels for studying epithelial and smooth muscle cell interactions. *Lab Chip* 18 1298–1309. 10.1039/c7lc01357d 29651473

[B42] Jaffar Z. Roberts K. Pandit A. Linsley P. Djukanovic R. Holgate S. (1999). B7 costimulation is required for IL-5 and IL-13 secretion by bronchial biopsy tissue of atopic asthmatic subjects in response to allergen stimulation. *Am. J. Respir. Cell Mol. Biol.* 20 153–162. 10.1165/ajrcmb.20.1.3255 9870929

[B43] Jain A. Barrile R. Van Der Meer A. D. Mammoto A. Mammoto T. De Ceunynck K. (2018). Primary human lung alveolus-on-a-chip model of intravascular thrombosis for assessment of therapeutics. *Clin. Pharmacol. Ther.* 103 332–340. 10.1002/cpt.742 28516446 PMC5693794

[B44] Klameth L. Rath B. Hochmaier M. Moser D. Redl M. Mungenast F. (2017). Small cell lung cancer: model of circulating tumor cell tumorospheres in chemoresistance. *Sci. Rep.* 7:5337. 10.1038/s41598-017-05562-z PMC5509650 28706293

[B45] Kolesky D. B. Truby R. L. Gladman A. S. Busbee T. A. Homan K. A. Lewis J. A. (2014). 3D bioprinting of vascularized, heterogeneous cell-laden tissue constructs. *Adv. Mater.* 26 3124–3130. 10.1002/adma.201305506 24550124

[B46] Konishi S. Gotoh S. Tateishi K. Yamamoto Y. Korogi Y. Nagasaki T. (2016). Directed induction of functional multi-ciliated cells in proximal airway epithelial spheroids from human pluripotent stem cells. *Stem Cell Rep.* 6 18–25. 10.1016/j.stemcr.2015.11.010 26724905 PMC4720023

[B47] Lang D. S. Droemann D. Schultz H. Branscheid D. Martin C. Ressmeyer A. R. (2007). A novel human ex vivo model for the analysis of molecular events during lung cancer chemotherapy. *Respir. Res.* 8:43. 10.1186/1465-9921-8-43 PMC1913052 17567922

[B48] Lauenstein L. Switalla S. Prenzler F. Seehase S. Pfennig O. Forster C. (2014). Assessment of immunotoxicity induced by chemicals in human precision-cut lung slices (PCLS). *Toxicol. In Vitro* 28 588–599. 10.1016/j.tiv.2013.12.016 24412833

[B49] Lenz A. G. Karg E. Lentner B. Dittrich V. Brandenberger C. Rothen-Rutishauser B. (2009). A dose-controlled system for air-liquid interface cell exposure and application to zinc oxide nanoparticles. *Part. Fibre Toxicol.* 6:32. 10.1186/1743-8977-6-32 20015351 PMC2804607

[B50] Lewis K. J. R. Hall J. K. Kiyotake E. A. Christensen T. Balasubramaniam V. Anseth K. S. (2018). Epithelial-mesenchymal crosstalk influences cellular behavior in a 3D alveolus-fibroblast model system. *Biomaterials* 155 124–134. 10.1016/j.biomaterials.2017.11.008 29175081 PMC5748390

[B51] Liu G. Betts C. Cunoosamy D. M. Aberg P. M. Hornberg J. J. Sivars K. B. (2019). Use of precision cut lung slices as a translational model for the study of lung biology. *Respir. Res.* 20:162. 10.1186/s12931-019-1131-x PMC6642541 31324219

[B52] Liu R. An L. Liu G. Li X. Tang W. Chen X. (2015). Mouse lung slices: an *ex vivo* model for the evaluation of antiviral and anti-inflammatory agents against influenza viruses. *Antiviral Res.* 120 101–111. 10.1016/j.antiviral.2015.05.008 26022197 PMC7125926

[B53] Lordan J. L. Davies D. E. Wilson S. J. Dent G. Corkhill A. Jaffar Z. (2001). The role of CD28-B7 costimulation in allergen-induced cytokine release by bronchial mucosa from patients with moderately severe asthma. *J. Allergy Clin. Immunol.* 108 976–981. 10.1067/mai.2001.119740 11742276

[B54] Mccauley K. B. Hawkins F. Serra M. Thomas D. C. Jacob A. Kotton D. N. (2017). Efficient derivation of functional human airway epithelium from pluripotent stem cells via temporal regulation of Wnt signaling. *Cell Stem Cell* 20 844–857.e6. 10.1016/j.stem.2017.03.001 28366587 PMC5457392

[B55] Meenach S. A. Tsoras A. N. Mcgarry R. C. Mansour H. M. Hilt J. Z. Anderson K. W. (2016). Development of three-dimensional lung multicellular spheroids in air- and liquid-interface culture for the evaluation of anticancer therapeutics. *Int. J. Oncol.* 48 1701–1709. 10.3892/ijo.2016.3376 26846376 PMC4777598

[B56] Miller A. J. Dye B. R. Ferrer-Torres D. Hill D. R. Overeem A. W. Shea L. D. (2019). Generation of lung organoids from human pluripotent stem cells *in vitro*. *Nat. Protoc.* 14 518–540. 30664680 10.1038/s41596-018-0104-8 PMC6531049

[B57] Miller A. J. Hill D. R. Nagy M. S. Aoki Y. Dye B. R. Chin A. M. (2018). *In vitro* induction and *in vivo* engraftment of lung bud tip progenitor cells derived from human pluripotent stem cells. *Stem Cell Rep.* 10 101–119. 10.1016/j.stemcr.2017.11.012 29249664 PMC5770275

[B58] Miller A. J. Spence J. R. (2017). *In vitro* models to study human lung development, disease and homeostasis. *Physiology* 32 246–260. 10.1152/physiol.00041.2016 28404740 PMC6148341

[B59] Neuhaus V. Schaudien D. Golovina T. Temann U. A. Thompson C. Lippmann T. (2017). Assessment of long-term cultivated human precision-cut lung slices as an ex vivo system for evaluation of chronic cytotoxicity and functionality. *J. Occup. Med. Toxicol.* 12:13. 10.1186/s12995-017-0158-5 PMC5446749 28559920

[B60] Nicholas B. Staples K. J. Moese S. Meldrum E. Ward J. Dennison P. (2015). A novel lung explant model for the ex vivo study of efficacy and mechanisms of anti-influenza drugs. *J. Immunol.* 194 6144–6154. 10.4049/jimmunol.1402283 25934861 PMC4456633

[B61] Niemeyer B. F. Zhao P. Tuder R. M. Benam K. H. (2018). Advanced microengineered lung models for translational drug discovery. *SLAS Discov.* 23 777–789. 10.1177/2472555218760217 29447055

[B62] Porzionato A. Stocco E. Barbon S. Grandi F. Macchi V. De Caro R. (2018). Tissue-engineered grafts from human decellularized extracellular matrices: a systematic review and future perspectives. *Int. J. Mol. Sci.* 19:4117. 10.3390/ijms19124117 30567407 PMC6321114

[B63] Prunieras M. Regnier M. Woodley D. (1983). Methods for cultivation of keratinocytes with an air-liquid interface. *J. Invest. Dermatol.* 81 28s–33s. 6190962 10.1111/1523-1747.ep12540324

[B64] Quantius J. Schmoldt C. Vazquez-Armendariz A. I. Becker C. El Agha E. Wilhelm J. (2016). Influenza virus infects epithelial stem/progenitor cells of the distal lung: impact on fgfr2b-driven epithelial repair. *PLoS Pathog.* 12:e1005544. 10.1371/journal.ppat.1005544 27322618 PMC4913929

[B65] Rackley C. R. Stripp B. R. (2012). Building and maintaining the epithelium of the lung. *J. Clin. Invest.* 122 2724–2730. 10.1172/jci60519 22850882 PMC3408736

[B66] Sachs N. Papaspyropoulos A. Zomer-Van Ommen D. D. Heo I. Bottinger L. Klay D. (2019). Long-term expanding human airway organoids for disease modeling. *EMBO J.* 38:e100300. 10.15252/embj.2018100300 PMC6376275 30643021

[B67] Sanderson M. J. (2011). Exploring lung physiology in health and disease with lung slices. *Pulm. Pharmacol. Ther.* 24 452–465. 10.1016/j.pupt.2011.05.001 21600999 PMC3168687

[B68] Sato T. Morita M. Tanaka R. Inoue Y. Nomura M. Sakamoto Y. (2017). Ex vivo model of non-small cell lung cancer using mouse lung epithelial cells. *Oncol. Lett.* 63–6868. 10.3892/ol.2017.7098 PMC5754888 29344123

[B69] Skronska-Wasek W. Mutze K. Baarsma H. A. Bracke K. R. Alsafadi H. N. Lehmann M. (2017). Reduced frizzled receptor 4 expression prevents WNT/beta-catenin-driven alveolar lung repair in chronic obstructive pulmonary disease. *Am. J. Respir. Crit. Care Med.* 1.96 172–185. 10.1164/rccm.201605-0904oc 28245136

[B70] Stratmann A. T. Fecher D. Wangorsch G. Gottlich C. Walles T. Walles H. (2014). Establishment of a human 3D lung cancer model based on a biological tissue matrix combined with a Boolean in silico model. *Mol. Oncol.* 8 351–365. 10.1016/j.molonc.2013.11.009 24388494 PMC5528544

[B71] Sun H. Zhu Y. Pan H. Chen X. Balestrini J. L. Lam T. T. (2016). Netrin-1 regulates fibrocyte accumulation in the decellularized fibrotic sclerodermatous lung microenvironment and in bleomycin-induced pulmonary fibrosis. *Arthritis Rheumatol.* 68 1251–1261. 26749424 10.1002/art.39575 PMC5547894

[B72] Surolia R. Li F. J. Wang Z. Li H. Liu G. Zhou Y. (2017). 3D pulmospheres serve as a personalized and predictive multicellular model for assessment of antifibrotic drugs. *JCI Insight* 2:e91377. 10.1172/jci.insight.91377 PMC5256136 28138565

[B73] Thorne D. Adamson J. (2013). A review of *in vitro* cigarette smoke exposure systems. *Exp. Toxicol. Pathol.* 65 1183–1193. 10.1016/j.etp.2013.06.001 23850067

[B74] Toepke M. W. Beebe D. J. (2006). PDMS absorption of small molecules and consequences in microfluidic applications. *Lab Chip* 6 1484–1486. 17203151 10.1039/b612140c

[B75] Upadhyay S. Palmberg L. (2018). Air-liquid interface: relevant in vitro models for investigating air pollutant-induced pulmonary toxicity. *Toxicol. Sci.* 164 21–30. 10.1093/toxsci/kfy053 29534242

[B76] Van Der Velden J. L. Wagner D. E. Lahue K. G. Abdalla S. T. Lam Y. W. Weiss D. J. (2018). TGF-beta1-induced deposition of provisional extracellular matrix by tracheal basal cells promotes epithelial-to-mesenchymal transition in a c-Jun NH2-terminal kinase-1-dependent manner. *Am. J. Physiol. Lung Cell. Mol. Physiol.* 314 L984–L997. 29469614 10.1152/ajplung.00053.2017 PMC6032072

[B77] Vijayanand P. Durkin K. Hartmann G. Morjaria J. Seumois G. Staples K. J. (2010). Chemokine receptor 4 plays a key role in T cell recruitment into the airways of asthmatic patients. *J. Immunol.* 184 4568–4574. 10.4049/jimmunol.0901342 20237293

[B78] Williamson I. A. Arnold J. W. Samsa L. A. Gaynor L. Disalvo M. Cocchiaro J. L. (2018). A high-throughput organoid microinjection platform to study gastrointestinal microbiota and luminal physiology. *Cell. Mol. Gastroenterol. Hepatol.* 6 301–319. 10.1016/j.jcmgh.2018.05.004 30123820 PMC6092482

[B79] Woodruff P. G. Innes A. L. (2006). Quantitative morphology using bronchial biopsies. *Eur. Respir. Rev.* 15 157–161. 10.1183/09059180.00010106

[B80] World Health Organization [WHO] (2018). *The Top 10 Causes of Death.* Available online at: https://www.who.int/news-room/fact-sheets/detail/the-top-10-causes-of-death (accessed March 31, 2020).

[B81] Yamaya M. Finkbeiner W. Chun S. Widdicombe J. (1992). Differentiated structure and function of cultures from human tracheal epithelium. *Am. J. Physiol. Lung Cell. Mol. Physiol.* 262 L713–L724. 10.1152/ajplung.1992.262.6.L713 1616056

[B82] Zacharias W. J. Frank D. B. Zepp J. A. Morley M. P. Alkhaleel F. A. Kong J. (2018). Regeneration of the lung alveolus by an evolutionarily conserved epithelial progenitor. *Nature* 555 251–255. 10.1038/nature25786 29489752 PMC6020060

[B83] Zhou Y. Horowitz J. C. Naba A. Ambalavanan N. Atabai K. Balestrini J. (2018). Extracellular matrix in lung development, homeostasis and disease. *Matrix Biol.* 73 77–104. 29524630 10.1016/j.matbio.2018.03.005 PMC6129220

